# Ecological Roles of Biodegradable Mulch Film on Soil Nutrients and Microbes

**DOI:** 10.3390/biology15120929

**Published:** 2026-06-15

**Authors:** Jie Wang, Xueyun Zheng, Yi Wei, Jinlin Xia, Fuwei Wang, Jianrong Zhao, Xiaoliang Li, Jianfei Wang

**Affiliations:** 1 College of Resources and Environment, Anhui Science and Technology University, Chuzhou 233100, China; 2 Dangtu County Shengnong Agriculture Technology Co., Ltd., Maanshan 243000, China; 3 Anhui Province Agricultural Waste Fertilizer Utilization and Cultivated Land Quality Improvement Engineering Research Center, Chuzhou 233100, China

**Keywords:** biodegradable mulch film, ecological roles, degradation, soil microbes, soil nutrients

## Abstract

Biodegradable mulch film (BDM) is increasingly promoted as a sustainable alternative to conventional polyethylene mulch film because it can degrade in soil after agricultural use. However, current evaluations mainly focus on degradation efficiency and crop productivity, often assuming that biodegradation automatically implies ecological safety. Here, we argue that this assumption is incomplete. Rather than acting as a passive substitute, BDM degradation represents a temporally structured ecological disturbance that actively alters soil microbial metabolism, extracellular enzyme stoichiometry, nutrient cycling, and rhizosphere function. We propose that the ecological consequences of BDM are primarily determined by whether polymer degradation is synchronized with microbial succession and crop nutrient demand. This perspective shifts the evaluation of BDM from degradation efficiency and economic benefit toward ecosystem functionality and provides a new theoretical framework for designing next-generation biodegradable agricultural materials.

## Simple Summary

As an eco-friendly material, biodegradable mulch film (BDM) is increasingly being adopted in agricultural production, offering an effective solution for sustainable development. The biodegradation process not only concerns the degradation efficiency of the mulch films themselves but may also have a profound impact on the structure and function of soil microbial communities, as well as the cycling and availability of soil nutrients. Here, we critically re-evaluate the ecological roles of BDM, providing a scientific basis beyond simple “waste reduction” for its true consequences on soil microbes and nutrient cycling.

## 1. Introduction

Polyethylene mulch film (PEMF) has become an essential agricultural technology because of its significant benefits in soil temperature regulation, water conservation, weed suppression, and crop productivity enhancement [1,2]. However, long-term application of PEMF has resulted in severe accumulation of plastic residues in agricultural soils, causing “white pollution”, deterioration of soil structure, obstruction of water and nutrient transport, and potential risks to terrestrial ecosystems and food security [3,4]. The increasing concern regarding agricultural plastic pollution has accelerated the development and adoption of biodegradable mulch film (BDM) as an environmentally friendly alternative [5,6,7,8].

BDM is commonly manufactured using biodegradable polymers such as starch, cellulose, polylactic acid (PLA), polyhydroxyalkanoates (PHA), and polybutylene adipate-co-terephthalate (PBAT) [5,6,7,8]. Unlike conventional polyethylene films, BDM can be fragmented and metabolized by soil microorganisms after field application, eventually forming carbon dioxide (CO_2_), water, and microbial biomass under appropriate environmental conditions [9,10,11]. Consequently, BDM is widely regarded as a sustainable solution that can reduce long-term plastic pollution while maintaining agricultural benefits [12,13,14].

However, current evaluations of the ecological impacts of BDM remain largely insufficient. Most studies primarily focus on short-term responses in degradation efficiency, residue losses, and crop yields [15,16,17,18]. This framework implicitly assumes that biodegradation itself equates to ecological compatibility. We argue that this assumption is conceptually incomplete because the degradation of BDM is not an environmentally neutral process. Instead, BDM degradation introduces exogenous carbon (C) substrates, alters soil microbial metabolism, extracellular enzyme stoichiometry, nutrient cycling, and rhizosphere function.

More importantly, these ecological responses are highly dynamic and phase-dependent. During degradation, BDM continuously modifies the soil microenvironment through C release, microbial stimulation, enzymatic activation, and accumulation of degradation intermediates [19,20,21,22]. These processes can generate transient nutrient immobilization, microbial functional specialization, and long-term legacy effects that extend beyond visible film disappearance [23,24]. Thus, the ecological impact of BDM may depend less on whether the material degrades and more on whether the timing of degradation is synchronized with microbial succession and crop nutrient demand.

Here, we propose a new ecological perspective in which BDM degradation is conceptualized as a temporally structured ecological disturbance process. We further introduce the concept of “temporal mismatch” to explain how asynchronous interactions among polymer decomposition, microbial metabolism, and plant nutrient acquisition determine the ecological outcomes of BDM application. By shifting the focus from material degradation toward ecosystem functionality, this article aims to establish a mechanistic ecological framework for understanding BDM–soil interactions.

## 2. Current Evaluation Frameworks of BDM Degradation

Existing studies typically assess BDM performance using indicators such as degradation rate, residue disappearance, tensile strength, soil coverage efficiency, and crop yield improvement [15,16,17,18]. Although these indicators are important for practical agricultural applications, they provide only limited insight into the ecological consequences of BDM degradation.

A major limitation of the current framework is the implicit assumption that faster degradation necessarily indicates better environmental performance. In reality, rapid degradation may induce intense microbial competition for nutrients, causing transient nitrogen (N) and phosphorus (P) immobilization during critical crop growth periods [25,26,27,28,29,30]. Conversely, excessively slow degradation may prolong physical soil disturbance, delay rhizosphere recovery, and increase the accumulation of persistent oligomers and microplastic-like intermediates.

Another limitation is that most field studies evaluate only short-term ecological responses. Current experiments commonly span one or two growing seasons and therefore fail to capture long-term ecological legacy effects associated with repeated BDM application. Soil microbial communities possess strong ecological functions, and repeated exposure to polymer-derived substrates may gradually alter microbial network stability, functional redundancy, and resilience [31,32,33,34].

Furthermore, most studies treated BDM as a homogeneous material category despite substantial differences among polymer types. Natural polymer-based films, such as starch-containing materials, generally undergo rapid hydrolysis and release labile C substrates [35,36]. In contrast, synthetic biodegradable polymers such as PLA and PBAT degrade more slowly and may generate persistent acidic monomers and oligomers [37,38,39]. These divergent degradation pathways likely produce fundamentally different microbial succession and nutrient cycling responses.

Current frameworks also largely overlook rhizosphere-scale interactions, and degrading polymers simultaneously compete for C, N, P, oxygen, and microsites within the rhizosphere [25,37,40]. Consequently, BDM degradation should not be viewed as an isolated decomposition process but rather as a dynamic ecological interaction embedded within the soil–plant–microbe system.

We therefore argue that the current material-centered evaluation paradigm is insufficient for assessing the true sustainability of BDM degradation. A mechanistic ecological framework is needed to understand how degradation dynamics interact with microbial metabolism, nutrient allocation, and plant development over time.

## 3. Biodegradable Mulch Film Degradation as a Temporally Structured Ecological Disturbance Process

**Figure 1:**
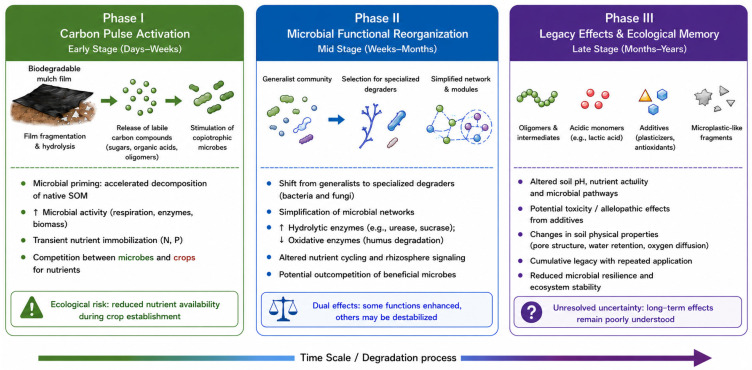
Conceptual framework: Biodegradable mulch film (BDM) degradation as a temporally structured ecological disturbance process. Upward and downward arrows represent positive and negative effects, respectively.

We propose that BDM degradation should be conceptualized as a temporally structured ecological disturbance process. This disturbance process consists of sequential but overlapping ecological phases (Figure 1). Unlike conventional views that consider biodegradation as a simple material disappearance process, this framework emphasizes that BDM actively reorganizes microbial metabolism and nutrient cycling throughout degradation.

### 3.1. Phase I: Carbon Pulse Activation

The initial stage of BDM degradation is characterized by physical fragmentation and early enzymatic hydrolysis [10,18]. During this phase, low-molecular-weight C compounds, including sugars, organic acids, and oligomers, are released into the soil environment. These compounds act as labile C substrates that rapidly stimulate copiotrophic microorganisms. This sudden C influx can trigger a microbial priming effect, in which soil microorganisms accelerate the decomposition of native soil organic matter to obtain limiting nutrients such as N and P [41,42]. Consequently, microbial respiration, extracellular enzyme activity, and microbial biomass often increase substantially during the early degradation phase [42]. However, this process may also induce transient nutrient immobilization. Because microbial growth requires balanced stoichiometric nutrient acquisition, microorganisms stimulated by C-rich substrates may rapidly consume available soil N and P for biomass synthesis [43,44]. Under nutrient-limited conditions, crops and microorganisms therefore compete directly for available nutrients. We argue that this transient nutrient competition represents one of the most overlooked ecological risks of BDM. Rapid early-stage degradation may temporarily reduce nutrient availability precisely during critical crop establishment periods, potentially offsetting agronomic benefits associated with mulching.

### 3.2. Phase II: Microbial Functional Reorganization

Along with the degradation progressing, microbial communities become increasingly structured around specialized degradative functions. The soil microbiome shifts from generalist resource utilization toward metabolic specialization driven by polymer-derived substrates. Natural polymer-based BDM typically favors fast-growing bacterial taxa capable of utilizing starch- or cellulose-derived C compounds. In contrast, synthetic polymers such as PLA and PBAT often select for specialized fungal and bacterial degraders capable of metabolizing complex polyester structures [45,46]. These differences generate distinct microbial succession trajectories. At the community level, BDM degradation may alter microbial co-occurrence network topology. Previous studies indicate that biodegradable polymers can simplify microbial interaction networks by reducing functional redundancy and promoting specialized ecological modules [22,28,34]. Over time, this functional reorganization may alter soil nutrient mineralization pathways, rhizosphere signaling processes, and ecosystem stability. Importantly, microbial stimulation does not necessarily imply ecological improvement. Specialized polymer degraders may outcompete beneficial rhizosphere microorganisms, including arbuscular mycorrhizal fungi and plant growth-promoting bacteria [33,34]. Thus, microbial activation induced by BDM may simultaneously enhance certain ecosystem functions while destabilizing others. During the degradation, BDM-derived C substrates fundamentally alter soil extracellular enzyme dynamics, driving significant shifts in enzymatic stoichiometry and microbial resource allocation. During this process, the microbial community adjusts its metabolic investment by upregulating specific enzyme systems to access the newly available polymer-derived C while mining for limiting nutrients [29,35]. Specifically, C-acquiring enzymes (such as cellulase, amylase, and β-glucosidase) are expected to be stimulated to facilitate the hydrolysis of polymer fragments [29,35]. Simultaneously, because most BDM polymers are C-rich but N- and P-poor, microbial growth on these substrates creates a stoichiometric imbalance. To meet their heightened N and P demands, microorganisms may increase the production of N-acquiring enzymes (such as urease, protease, and leucine aminopeptidase) and P-acquiring enzymes (such as alkaline phosphatase), a phenomenon indicative of microbial N and P mining from native soil organic matter [20,42,46]. Conversely, oxidative enzymes associated with the degradation of recalcitrant humus (such as phenol oxidase and peroxidase) frequently decline [29]. This enzymatic trade-off reflects a strategic shift in microbial metabolism. The readily labile C from BDM reduces the energy-requiring need to degrade stable soil organic matter, temporarily suppressing humus mineralization [35,41]. Over time, this altered enzyme stoichiometry not only modifies the pathways of soil nutrient mineralization but also creates a transient decoupling between C and nutrient cycling, which can persist until the polymer substrates are exhausted.

### 3.3. Phase III: Legacy Effects and Ecological Memory

The final phase of BDM degradation involves the accumulation and persistence of degradation intermediates, oligomers, additives, and microplastic-like fragments. Although visible film residues may disappear, ecological recovery may not occur immediately. Synthetic biodegradable polymers frequently produce acidic monomers and intermediate compounds during degradation. For example, PLA degradation releases lactic acid, while PBAT degradation can generate terephthalic acid derivatives [11,12,39,43]. These compounds may alter soil pH, nutrient solubility, and microbial metabolic pathways. Furthermore, additives incorporated into BDM formulations, including plasticizers, antioxidants, and stabilizers, may exert toxicological or allelopathic effects on non-target organisms [22,24,28]. Persistent oligomers and microplastic intermediates may also physically modify soil pore structure, water retention characteristics, and oxygen diffusion [8,21,41]. Repeated annual application of BDM may therefore generate cumulative ecological legacy effects. Over multiple growing seasons, microbial communities may gradually shift toward communities enriched in specialized degraders, which could potentially reduce microbial functional redundancy and ecosystem resilience. We propose that these long-term legacy effects represent one of the largest unresolved uncertainties in current BDM application research.

## 4. Temporal Mismatch Between BDM Degradation and Crop Nutrient Demand

**Figure 2:**
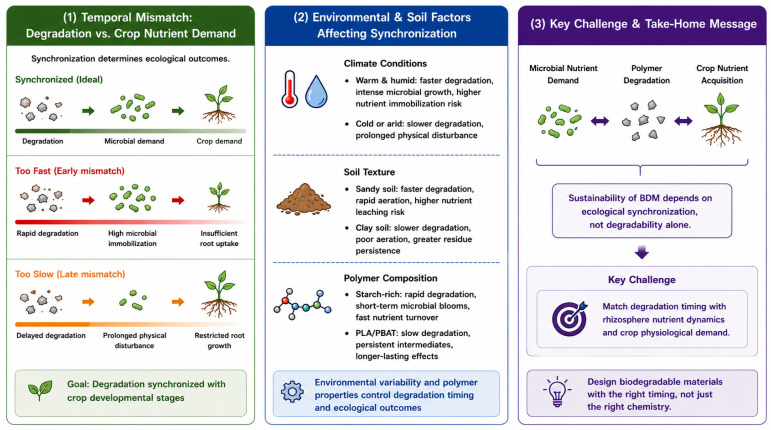
Conceptual framework for the temporal mismatch between BDM degradation and plant growth.

We further propose that the ecological consequences of BDM are primarily governed by the degree of temporal synchronization among polymer degradation, microbial nutrient demand, and crop nutrient acquisition (Figure 2). Under ideal conditions, BDM degradation should occur gradually and remain synchronized with crop developmental stages. Early-stage degradation should not induce severe nutrient immobilization during seedling establishment, whereas late-stage degradation should not physically constrain root expansion or water movement. However, environmental variability often disrupts this synchronization. In warm and humid soils, rapid degradation may stimulate intense microbial growth and nutrient immobilization before crops establish sufficient root systems [40,42,45]. Conversely, in cold or arid soils, delayed degradation may prolong physical disturbance and reduce ecological transition efficiency [39,46]. Soil texture further modifies this process. Sandy soils typically exhibit faster aeration and microbial turnover, resulting in rapid degradation but higher nutrient leaching risk. Clay soils often slow degradation due to reduced oxygen diffusion and stronger physical protection of polymer fragments, potentially increasing residue persistence [18,35,44]. Importantly, different polymer compositions exhibit highly distinct degradation kinetics. Starch-rich materials frequently generate short-term microbial blooms and rapid nutrient turnover, whereas PLA/PBAT systems may produce prolonged degradation trajectories associated with persistent intermediates [6,11,37].

We therefore argue that the sustainability of BDM application depends not simply on degradability but on ecological synchronization. The key challenge for future biodegradable agricultural materials is to match degradation timing with rhizosphere nutrient dynamics and crop physiological demand.

## 5. Toward a Mechanistic Ecological Framework of Biodegradable Mulch Film

Future BDM research should move beyond degradation characterization and establish a mechanistic ecological framework integrating soil microbiology, rhizosphere ecology, and biogeochemistry. First, long-term field experiments are urgently needed. Most current studies are limited to short experimental duration and therefore cannot capture cumulative ecological legacy effects associated with repeated BDM application [23,24,25]. Second, future studies should integrate multi-omics approaches, including metagenomics, metatranscriptomics, metabolomics, and proteomics, to reveal how microbial functional pathways respond dynamically to polymer degradation. Third, stable isotope tracing techniques should be employed to distinguish C and nutrient fluxes originating from BDM degradation versus native soil organic matter. Such approaches would clarify the mechanistic basis of microbial priming and nutrient competition. Fourth, rhizosphere-scale monitoring is essential. Future studies should investigate how degrading polymers influence root exudation, microbial recruitment, nutrient hotspots, and plant–microbe signaling pathways. Finally, we propose that future biodegradable agricultural materials should be designed according to ecological synchronization principles rather than degradation speed alone. The next generation of BDM should optimize degradation timing, nutrient release dynamics, microbial compatibility, and rhizosphere stability simultaneously. This perspective fundamentally shifts biodegradable material design from polymer engineering toward ecological engineering.

## 6. Conclusions

The “temporal mismatch” framework provides a new conceptual basis for evaluating biodegradable agricultural materials. We propose that rapid degradation of BDM is not necessarily environmentally beneficial, and visible residue disappearance does not guarantee ecological recovery. Instead, transient nutrient immobilization, shifts in soil enzyme stoichiometry and enzymatic trade-offs, microbial functional reorganization, and long-term legacy effects collectively determine the true sustainability of BDM. The ecological outcome of BDM may be largely determined by the temporal synchronization among polymer decomposition, microbial nutrient demand, and crop nutrient acquisition.

Future studies integrating long-term field experiments, stable isotope tracing, microbial network analysis, and multi-omics approaches will be essential for establishing a predictive ecological theory of BDM–soil interactions. Ultimately, the next generation of biodegradable agricultural materials should be designed not merely for degradability, but for ecological compatibility and rhizosphere synchronization.

## Data Availability

No new data were created or analyzed in this study. Data sharing is not applicable.

## References

[1] He W., Yan C., Liu Q. (2019). Effects of polyethylene mulch residue on soil physicochemical properties and crop yield. Acta Ecol. Sin..

[2] Barnes D.K.A., Galgani F., Thompson R.C., Barlaz M. (2019). Accumulation and fragmentation of plastic debris in global environments. Philos. Trans. R. Soc. B.

[3] Chae Y., An Y.J. (2018). Current research trends on plastic pollution and ecological impacts on the soil ecosystem: A review. Environ. Pollut..

[4] de Souza Machado A.A., Kloas W., Zarfl C., Hempel S., Rillig M.C. (2018). Microplastics as an emerging threat to terrestrial ecosystems. Glob. Change Biol..

[5] Luckachan G.E., Pillai C.K.S. (2011). Biodegradable polymers-a review on recent trends and emerging perspectives. J. Polym. Environ..

[6] Wang Z., Su Y., Jiang Z. (2021). Advances in the synthesis, modification, and application of PBAT biodegradable polyesters. Chem. Ind. Prog..

[7] Sivan A. (2011). New perspectives in plastic biodegradation. Curr. Opin. Biotechnol..

[8] Emadian S.M., Onay T.T., Demirel B. (2017). Biodegradation of bioplastics in natural environments. Waste Manag..

[9] Zheng W., Wang L., Chen G. (2020). Advances in the biosynthesis and applications of polyhydroxyalkanoates (PHAs). Chin. J. Biotechnol..

[10] Li X., Zheng G., Li Z., Fu P. (2024). Formulation, performance and environmental/agricultural benefit analysis of biomass-based biodegradable mulch films: A review. Eur. Polym. J..

[11] Ryu Y., Bouharras F.E., Cha M., Mudondo J., Kim Y., Ramakrishnan S.R., Shin S., Yu Y., Lee W., Park J. (2025). Recent advancements in the evolution, production, and degradation of biodegradable mulch films: A review. Environ. Res..

[12] Chinaglia S., Tosin M., Degli-Innocenti F. (2019). Biodegradation rate of biodegradable plastics at molecular level. Polym. Degrad. Stab..

[13] Xiong L., Li Z., Shah F., Wang P., Yuan Q., Wu W. (2024). Biodegradable mulch film enhances the environmental sustainability compared with traditional polyethylene film from multidimensional perspectives. Chem. Eng. J..

[14] Liu S., Jin R., Li T., Yang S., Shen M. (2024). Are biodegradable plastic mulch films an effective way to solve residual mulch film pollution in farmland?. Plant Soil.

[15] Hayes D.G., Anunciado M.B., DeBruyn J.M., Bandopadhyay S., Schaeffer S., English M., Ghimire S., Miles C., Flury M., Sintim H.Y., Gutiérrez T. (2019). Biodegradable plastic mulch films for sustainable specialty crop production. Polymers for Agri-Food Applications.

[16] Akhir M.A.M., Mustapha M. (2022). Formulation of biodegradable plastic mulch film for agriculture crop protection: A review. Polym. Rev..

[17] Haider T.P., Völker C., Kramm J., Landfester K., Wurm F.R. (2019). Plastics of the future? The impact of biodegradable polymers on the environment and on society. Angew. Chem..

[18] Qi R., Jones D.L., Liu Q., Liu Q., Li Z., Yan C. (2021). Field test on the biodegradation of PBAT-based mulch films in soil. Polym. Test..

[19] Sintim H.Y., Flury M. (2017). Is biodegradable plastic mulch the solution to agriculture’s plastic problem?. Environ. Sci. Technol..

[20] Rillig M.C., Leifheit E., Lehmann A. (2021). Microplastic effects on carbon cycling processes in soils. PLoS Biol..

[21] Piao Z., Agyei Boakye A.A., Yao Y. (2024). Environmental impacts of biodegradable microplastics. Nat. Chem. Eng..

[22] Zhang H., Shu D., Zhang J., Liu X., Wang K., Jiang R. (2024). Biodegradable film mulching increases soil microbial network complexity and decreases nitrogen-cycling gene abundance. Sci. Total Environ..

[23] Graf M., Greenfield L.M., Reay M.K., Bargiela R., Golyshin P.N., Evershed R.P., Lloyd C.E.M., Williams G.B., Chadwick D.R., Jones D.L. (2024). Field-based assessment of the effect of conventional and biodegradable plastic mulch film on nitrogen partitioning, soil microbial diversity, and maize biomass. Appl. Soil Ecol..

[24] Liu X., Wen Z., Zhou W., Dong W., Ren H., Liang G., Gong W. (2025). Effect of multiyear biodegradable plastic mulch on soil microbial community assembly and functioning. Microorganisms.

[25] Qi Y., Ossowicki A., Yang X., Lwanga E.H., Dini-Andreote F., Geissen V., Garbeva P. (2020). Effects of plastic mulch film residues on wheat rhizosphere and soil properties. J. Hazard. Mater..

[26] Kuzyakov Y., Xu X. (2013). Competition between roots and microorganisms for nitrogen: Mechanisms and ecological relevance. New Phytol..

[27] Paliaga S., Badalucco L., Ciaramitaro V.C., Martino D.F.C., Gelsomino A., Kandeler E., Marhan S., Laudicina V.A. (2025). Fertilizer enriched bio-based mulch films increase nitrogen and phosphorus availability and stimulate soil microbial biomass and activity. Appl. Soil Ecol..

[28] Liu S.E., Zhang A., Guo H., Min W. (2026). Biodegradable mulch enhances cotton (*Gossypium hirsutum* L.) yield via microplastic reduction and P cycling microbes modulation. Field Crops Res..

[29] Li S., Wang X., Li T. (2020). Effects of biodegradable mulch films on soil nitrogen, phosphorus, and potassium nutrients and enzyme activities. J. Plant Nutr. Fertil. Sci..

[30] Scopetani C., Pellinen J., Selonen S. (2024). Phthalates and other organic chemicals in agricultural soils after use of different types of conventional and biodegradable plastics. Environ. Res..

[31] Bandopadhyay S., Sintim H.Y., DeBruyn J.M. (2020). Effects of biodegradable plastic film mulching on soil microbial communities in two agroecosystems. PeerJ.

[32] Santini G., Probst M., Gómez-Brandón M., Manfredi C., Ceccherini M.T., Pietramellara G., Santorufo L., Maisto G. (2024). Microbiome dynamics of soils covered by plastic and bioplastic mulches. Biol. Fertil. Soils..

[33] Liu L., Li L., Zou G., Gu J., Zuo Q., Zheng X., Du L., Liu D. (2025). Soil fungi respond more violently to both polyethylene and PBAT biodegradable mulch film residues than bacteria do. Front. Environ. Sci..

[34] Wei M., Wang Y., Xie F., Sun Q., Shao H., Cheng X., Wang X., Tao X., He X., Yong B. (2025). The ecological trap: Biodegradable mulch film residue undermines soil fungal network stability. Microorganisms.

[35] Huo Y., Dijkstra F.A., Possell M., Singh B. (2024). Mineralisation and priming effects of a biodegradable plastic mulch film in soils: Influence of soil type, temperature and plastic particle size. Soil Biol. Biochem..

[36] Zantis L.J., Straetemans S., Adamczyk S., Adamczyk B., Velmala S., Brandsma S., Margalef M., Bosker T. (2025). Breaking ground: The effect of leachates from conventional and biodegradable mulching films on plants. Environ. Chem. Ecotoxicol..

[37] Zhong J., Li J., Liao J., Ma Y., Li Z., Yang L., Chang W., Miao M. (2025). Alpine radish rhizosphere microbiome assembly and metabolic adaptation under PBAT/PLA humic acid biodegradable mulch films. Front. Microbiol..

[38] Zhu Y., Fu B., Zhu P., Shu S., Wang D., Xu H., Cai D. (2024). Waste milk-derived biodegradable mulch film fabrication and nutrient slow-release performance evaluation. Chem. Eng. J..

[39] Graf M., Choiselat E., Reay M.K., Bargiela R., Dimitriou A., Liu Q., Elias R.M., Golyshin P.N., Griffiths R., Chadwick D.R. (2025). Biodegradable mulch films exhibit slower-than-expected degradation with negligible effects on soil microbial communities. J. Hazard. Mater..

[40] Liu S., Hu X., Wang S., Guan Z., Yuan C., Tian C., Luo S. (2026). Biodegradable mulch films drive microbial divergence and heighten environmental risks. Appl. Soil Ecol..

[41] Ramanayaka S., Zhang H., Semple K.T. (2024). Environmental fate of microplastics and common polymer additives in non-biodegradable plastic mulch applied agricultural soils. Environ. Pollut..

[42] Zhang H., Yu S., Lv W., Wang X., Lu P., Zhang H., Zhang J., Bai N., Xu C., Zhu X. (2026). Biodegradable mulch films divergently regulate soil carbon cycle by reshaping microbial communities and functional genes. J. Hazard. Mater..

[43] Gao W., Tu Z., Yin X., Ming S., Cai K. (2025). Effects of PBAT biodegradable mulch on lettuce (*Lactuca sativa* L.) physiology and soil microbial community: Based on a long-term degradation trial. Ecotoxicol. Environ. Saf..

[44] Mazzon M., Edo C., Guerrini S., Gioacchini P., Cupi J., Malena P., Rosal R., Marzadori C. (2026). Long-term biodegradable mulch films application in agricultural fields: Effects on soil functionality and microplastic generation. J. Environ. Manag..

[45] Zhang D., Ng E.L., Hu W., Ding Y., Long M., Zhang G., Qin X., Siddique K.H.M., Liao Y. (2022). Response of soil microbial community parameters to plastic film mulch: A meta-analysis. Geoderma.

[46] Zhang H., Zhao J., Huang Y., Wang B., Liu R., Zhu Z., An S. (2025). Microplastics from conventional and biodegradable mulch films alter microbial necromass accumulation and organic carbon sequestration in farmland soils. Environ. Pollut..

